# Review of previously published ambient air respirable crystalline silica concentration data for use in risk assessment of mineral industry sources

**DOI:** 10.3389/fpubh.2025.1584841

**Published:** 2025-04-30

**Authors:** John R. Richards, Todd T. Brozell

**Affiliations:** Air Control Techniques, P.C., Cary, NC, United States

**Keywords:** ambient respirable crystalline silica concentrations, crystalline silica sampling methods, mineral industry crystalline silica emissions, chronic exposure concentrations, risk assessment

## Abstract

This paper is part of a set of papers concerning the possible health implications of ambient respirable crystalline silica beyond the fencelines of mineral industry sources such as frac sand, construction sand, crushed stone, and specialty silica product plants. Previously published ambient respirable crystalline silica data relevant to mineral industry sources are reviewed to identify the typical and maximum downwind ambient respirable crystalline silica concentrations expected beyond facility fencelines. These typical and maximum downwind concentrations can be used in risk assessment studies. Relevant RCS facility downwind and upwind ambient data sets previously compiled by the authors, state regulatory agencies, the Agency for Toxic Substances and Disease Registration (ASTDR), and academic researchers are reviewed. Emphasis is placed on data sets compiled in multi-year sampling programs that take into account seasonal variations and facility operational variations. To the extent possible, data from different geographic areas are included to account for differing background (upwind) concentrations. The ambient RCS data are condensed to yield mean and maximum long-term average concentrations that can be used in risk assessment studies. There is a high degree of consistency in the RCS concentration data published by numerous researchers at a wide variety of mineral industry facilities. The authors recommend a mean concentration of 0.28 μg/m^3^ as an estimate of the long term average RCS concentration downwind of the fencelines of typical mineral industry facilities and a maximum concentration of 1.5 μg/m^3^ as an estimate of 95% upper confidence level of the mean RCS concentration downwind of the fencelines of especially large mineral industry facilities and/or those facilities where the very hilly terrain limit dispersion of facility emissions. The authors recommend a mean concentration of 0.22 μg/m^3^ as an estimate of the typical long term average background concentrations. If there are community health concerns at the RCS concentrations in the range of 0.22 to 0.28 μg/m^3^, new measurement procedures that provide higher 24-h sample volumes and/or more sensitive analytical techniques would be needed to provide accurate community exposure data.

## 1 Introduction

This paper provides a summary of ambient respirable crystalline silica (RCS) concentration data in the vicinity of mineral industry sources. Later papers in this journal concern the potential health risks of community exposure in areas close to these sources.

The term RCS as used in this paper applies to data measured using ambient air samplers that have a 50% cut size for 4-micrometer diameter (aerodynamic diameter) and a size-efficiency curve similar to that for respirable particulate matter as stated in Table B2 in International Standards Organization (ISO) publication 7708-1995(E) (1). The terms RCS and PM4 respirable crystalline silica are used synonymously in this paper. Unless stated otherwise, all particle size data are expressed as aerodynamic diameters.

RCS data evaluated in this paper include quartz, cristobalite, and tridymite. Other crystalline silica polymorphs are too rare to be of interest and are not included in the most commonly applied analytical procedures used to quantify RCS.

The concentrations of RCS in ambient air around mineral industry facilities are of concern to nearby communities, regulatory agencies, and facility operators. Prior to 2005, few data were available to assess ambient RCS concentrations near quarries and mineral processing facilities. The ambient data that did exist were based on very limited crystalline silica data in particulate matter with a 50% cut size of 10 micrometers aerodynamic as measured by U.S. Environmental Protection Agency (EPA) reference methods (PM10) particulate matter. These PM10 crystalline silica content data were not directly comparable to the extensive dataset of occupational PM4 RCS concentrations compiled in numerous studies conducted over many years in many countries.

In 2005, the California Office of Environmental Health Hazard Assessment (OEHHA) adopted a chronic reference exposure level (REL) for RCS of 3.0 μg/m^3^ as a guideline to protect sensitive individuals from exposure to ambient respirable crystalline silica from natural, community, and industrial sources. OEHHA based this guideline primarily on five RCS occupational health studies summarized in OEHHA (2). This guideline was not adopted as an enforceable limit applicable to occupational exposures to RCS.

This paper reviews the published data to characterize the mean and maximum RCS ambient concentrations that can be used in risk analyses applicable to community areas around mineral industry facilities.

## 2 Methods

### 2.1 Ambient RCS sampling and analysis procedures

OEHHA based its REL on occupational hygiene epidemiological studies conducted using personal samplers designed for occupational exposures at concentrations much higher than ambient levels. The limit of quantification (LOQ) of the sampling methods specified in National Institute for Occupational Safety and Health (NIOSH) Manual of Analytical Methods (NMAM) (3, 4) used in most of the health effects studies is higher than appropriate for comparing ambient air exposure levels to the ambient air-oriented OEHHA REL. Different sampling methods for ambient RCS were needed to compare community exposures to this REL.

Prior to the development of OEHHA’s REL, Davis (5) had estimated ambient RCS concentrations in 22 U.S. cities based on crystalline silica analyses of PM2.5 filter samples obtained using dichotomous samplers. While the crystalline silica content in PM2.5 samples is logically slightly lower than in PM4 samples, his approach suggested that EPA sampling methods for ambient PM2.5 that inherently have higher sample volumes than occupational exposure respirable dust samplers could be used to measure ambient RCS. Furthermore, the Davis data indicated that the ambient RCS levels in the PM2.5 fraction were in the range of zero to 1.9 μg/m^3^ with a mean value of 0.36 μg/m^3^. Based on these data and the OEHHA REL, it became apparent that any new ambient RCS sampling approaches (1) should be based on modified EPA reference methods for PM2.5 particulate matter and (2) should have sufficient sample volume to allow for very low RCS LOQs.

In 2006, following the general approach used by Davis, Richards and Brozell developed a high volume sampling method for respirable crystalline based EPA’s PM2.5 sampler specified in 40 CFR Part 50 Appendix L. This method involved a relatively straightforward adjustment of the sample flowrate through the PM2.5 cyclone to achieve a 50% cut point of 4.0 micrometers (aerodynamic diameter) rather than 2.5 micrometers. Laboratory based tests using National Institute of Standards and Technology (NIST) traceable monodisperse microspheres indicated a 4.0 micrometer 50% cut size at a sample flowrate of 11 liters per minute. Rupprecht and Patashnick (6),^1^ the manufacturer of the Partisol 2000i PM2.5 sampler in 2006, confirmed this sample flowrate necessary to achieve the 50% cut size at 4 micrometers. This adjusted sample flowrate for PM4 particulate matter was 66% of the 17 liters per minute flowrate specified in Appendix L for PM2.5 particulate matter.

The EPA Appendix L PM2.5 samplers use a Teflon^®^ filter, which is not appropriate for the analysis of crystalline silica. Accordingly, the filter media used for PM4 particulate matter was changed to PVC to provide for NMAM 7500 analyses. Based on a 11 liter per minute flowrate and a total sample volume of 16 cubic meters in a 24-h sample, the LOQ using this sampling method combined with NMAM 7500 X-ray diffraction analyses is 0.31 micrograms per cubic meter. Accordingly, this sampling approach provides an effective means to measure PM4 particulate matter in a form that can be analyzed for the very low concentrations of crystalline silica present in ambient air.

Sampling with this Appendix L-based approach can be performed in full accordance with the comprehensive EPA (7, 8) quality assurance requirements for PM2.5 samplers. The scope of the quality assurance (QA) analyses includes (1) the use of collocated samplers to the extent reasonably possible, (2) monthly audits of the sample flow rates, filter and ambient temperatures, and pressures, (3) yearly three-point sample flow rate calibrations, (4) yearly ambient pressure, filter chamber pressure, ambient temperature, and filter chamber calibrations, (5) filter blank analyses of every tenth filter, and (6) review of the five-minute average sampler operating data as recorded by the samplers.

The South Coast Air Quality Management District (9, 10) developed an approach similar to that of Richards and Brozell for ambient crystalline sampling at Duarte, California. The Pennsylvania Department of Environmental Protection (DEP, 2016) used a similar ambient air sampling method in a study of crystalline silica concentrations near Tunkhannock, Pennsylvania (2017). The Minnesota Pollution Control Agency (11–13) used a sampling procedure based on the use of BGI PQ100 samplers that provides a slightly lower sample flowrate.

Most of the ambient RCS concentration data reviewed in this report were obtained by flow-adjusted EPA Appendix L-based PM4 samplers and NMAM 7500 XRD analyses.

Peters et al. (14) used a respirable dust sampler similar to the NMAM 7500 sampler but having a cyclone designed for a sample flowrate of 4.2 liters per minute and having a 50% cut size of 4 micrometers. The BGI GK2.69 cyclone sampler used by Peters et al. (14) had a PVC filter that can be analyzed using NMAM 7500 XRD. Sampling was conducted for 48 h to yield a LOQ of 0.4 micrograms per cubic meter.

This modified NMAM 7500 sampling approach used by Peters et al. provided high quality ambient respirable crystalline silica data; however, the 48-h sampling period limits the comparability of data with EPA, state, and local monitoring stations operating strictly on a 24-h basis. Considerable day-to-day variability can occur in the PM4 particulate matter concentrations.

### 2.2 Ambient RCS sampling site characteristics

EPA has specified numerous ambient air sampling requirements that differ from the requirements for occupational health exposure sampling. To obtain representative ambient air samples, the operating characteristics and the sampling locations for sampling programs near mineral industries (microscale site monitoring) should meet the following siting requirements of 40 CFR Part 58, Appendix E to the maximum extent possible.

- For industrial facility fenceline and other microscale applications, the sampler inlet should be 2–7 meters above the ground [40 CFR Part 58, Appendix E, Paragraph 2.1(d)].
- The ambient sampler should not be located directly on an unpaved, unvegetated surface (40 CFR Part 58, Appendix E, Paragraph 2.2).
- There should be unrestricted airflow in a 270 degree arc around the sampler (40 CFR Part 58, Appendix E, footnote 4 to Table E-3 of Section 2.7).
- No trees or shrubs should be between the sampler inlet and the source being evaluated. The sampler should be at least 10 meters from the dripline of the nearest tree [40 CFR Part 58, Appendix E, Paragraph 2.4(a)].
- If a roadway is not the main source of interest, the sampler should be 5–15 meters from the side of the nearest traffic lane [40 CFR Part 58, Appendix E, Paragraph 2.5.3 (b)].

While not required by EPA regulations, it is usually necessary to surround the sampler with a fence and locked gate to prevent damage due to animals and/or intentional tampering. In areas where there could be accumulation of mud or snow, a platform can be used without exceeding the 2–7 m sampler inlet height requirement. The ambient air samplers must have line electrical power or a substantial solar array and battery system to allow for operation during 24-h sampling periods.

Sampling is usually conducted on a once every third day or once every sixth day schedule. Sampling starts at midnight and continues for a full 24-h. Sampling should be performed on the specific calendar days specified by EPA each year to allow comparison of the data with ambient air quality data published by EPA, state, and local regulatory agencies.

### 2.3 Risk assessment study data selection criteria

The following criteria have been applied in selecting the ambient RCS concentration data that are most appropriate for use in risk assessment studies for communities near mineral industry facilities.

1. Sampling should have been conducted for PM4 particulate matter based on either flow-adjusted EPA PM2.5 ambient sampling procedures or the modified respirable dust sampler used by Peters et al. described in section 2.1 of this paper.
2. The sampling sites should have generally satisfied the EPA requirements specified in 40 CFR Part 58, Appendix E as described in section 2.2 of this paper.
3. The sampling quality assurance procedures should have been generally consistent with EPA quality assurance procedures for ambient PM2.5 particulate matter.
4. Analyses of crystalline silica should have included all three common polymorphs and have measurement LOQs equal to below or 0.40 μg/m^3^.
5. The sampling program should have been sufficiently long to take into account daily and seasonal variability in background concentrations and source operation variability.
6. Meteorological data should have been available to determine the dominant wind directions during sampling days.

These data set selection criteria exclude RCS data from very short term studies and data compiled using the numerous types of portable particulate matter samplers that do not provide samples that can be analyzed for crystalline silica. These criteria also exclude data obtained at inappropriate sampling locations or with the lack of important quality assurance procedures.

Consistent with the above six criteria, a large set of ambient RCS data has now been compiled by a variety of researchers including (1) ASTDR, (2) the South Coast Air Quality Management District, (3) the Minnesota Pollution Control Agency, (4) the Pennsylvania Department of Environmental Protection, (5) the University of Iowa, and (6) Richards and Brozell.

## 3 Ambient RCS monitoring data

### 3.1 Facility downwind concentration data

The published RCS concentration data downwind of mineral industry facilities are reviewed in this section to identify the typical and maximum ambient RCS levels that could be used for risk assessment. The published data are addressed in several regional groups with potentially different RCS background concentrations.

#### 3.1.1 Upper midwest area frac sand plants

Richards and Brozell (15, 16) conducted ambient RCS sampling studies at the downwind fencelines of 11 frac sand plants located in Wisconsin, Minnesota, and Illinois. The available data concern a wide variety of production rates, terrains, and community RCS source characteristics. Detailed information concerning the sampling sites, mineral industry sources, and sampling periods is available in the cited articles.

Many of these studies involved multi-year sampling programs that inherently took into account seasonal variations in background RCS levels and varying process operating conditions. Most studies included upwind-downwind sampling configurations around the mineral industry facilities. Most of these sampling programs also had a collocated respirable crystalline silica sampler at the downwind site to evaluate the precision of the RCS concentration data.

All of these sampling programs operated on the once-every-three day or once-every-sixth day sampling frequency specified by EPA. The sampling programs that included collocated samplers operated those samplers on a once-every-twelve-day schedule.

The crystalline silica content of the mineral materials handled at all of these frac sand facilities ranged from 95% to more than 99% by weight (17). This range exceeds the crystalline silica content of aggregate facilities handling granite, limestone, traprock and other common types of stone.

These Upper Midwest area studies included comprehensive quality assurance programs that satisfied and exceeded the EPA specified PM2.5 quality assurance procedures summarized in EPA ambient air sampling quality assurance documents (2013, 2017).

Forty to more than 90 % of the 24-h RCS concentrations measurements in all of the studies were below the limit of quantification (LOQ) of 0.31 μg/m^3^. For the numerous non-detect values, a value calculated as the LOQ/2 (0.16 μg/m^3^) was assigned. Considering that crystalline silica is present in most areas, this substitute value of 0.16 μg/m^3^ was more appropriate that a zero value for measurements below the LOQ.

The Upper Midwest area sampling programs summarized in Table 1 compiled more than 3,100 24-h ambient respirable crystalline silica measurements at facility downwind sites during a wide variety of frac sand facility operating conditions and a wide variety of weather conditions. The 95% upper confidence values of the mean were calculated based on nonparametric distributions.

**Table 1:** Upper Midwest frac sand plant sampling program results (16).

Facility	Type	Number of 24-h samples	Average with non-detects = LOQ/2 μg/m^3^	95% UCL^1^, mean RCS μg/m^3^
Kasota, Town	Processing plant and quarries	312	0.28	0.30
Kasota, Prairie		299	0.36	0.41
Kasota, 470st		318	0.42	0.49
Menomonie	Processing plant and quarry	63	0.23	0.27
Wedron	Processing plant and quarries	139	1.5	1.7
Sparta	Processing plant and quarries	419	0.24	0.25
Cataract Green	Quarry	108	0.18	0.20
Chippewa Falls	Processing plant	384	0.25	0.27
DS Quarry	Quarry	435	0.20	0.21
SS Quarry	Quarry	375	0.18	0.19
DD Quarry	Quarry	358	0.18	0.19
Maiden Rock	Processing plant and quarry	219	1.2	1.5
Downing	Quarry	63	0.22	0.25
Sample number weighted average			0.36	0.41
Total number of 24-h samples			3,492	

^195% UCLs were calculated using nonparametric distributions.^

All of the average values at downwind sites were in the range of 0.18–1.5 μg/m^3^. The weighted average downwind concentration for the set of Upper Midwest studies was 0.36 μg/m^3^. The 95% upper confidence interval of the mean was 0.41 μg/m^3^.

The highest downwind concentrations were measured at Wedron, Illinois, which is an especially large facility having many in-plant process units very close to the downwind sampling location.

The U.S. EPA and Agency for Toxic Substances and Disease Registration (18) conducted a separate ambient RCS sampling program at five downwind sampling locations in the Wedron community approximately 6 months after the conclusion of the Richards and Brozell sampling program. This program was conducted over a four-month period in late 2016. The mean RCS concentrations at the five downwind community sites ranged from 0.63 to 1.3 μg/m^3^. The 95% UCLs of the mean ranged from 0.7 to 1.6 μg/m^3^. These ASTDR reported values were very similar to those reported by Richards and Brozell (16) at the Wedron downwind site.

The Maiden Rock facility study had RCS downwind concentrations higher than most of the other Upper Midwest facility downwind monitoring locations. The Maiden Rock downwind samplers were in a narrow valley surrounded by high ridges on several sides of the plant. Some of the frac sand processing equipment and the plant access road were located very close to the downwind sampler inside the plant area.

The Wedron and Maiden Rock data sets provide a useful indication of the maximum downwind RCS concentrations that occur at especially large facilities and/or facilities located in hilly terrain, which limit plume dispersion.

#### 3.1.2 Western Wisconsin area frac sand plant study

Peters et al. (14) measured ambient PM4 respirable crystalline silica at 17 residences within 800 meters of the fencelines of quarries and other mineral processing facilities in Western Wisconsin. All 17 of the 48-h samples were below the reporting level of 0.4 μg/m^3.^ The authors concluded that the measured concentrations were below the level of concern with respect to the chronic reference exposure RELs in California and Minnesota. This paper did not include a tabulation of the results. Accordingly, the data have been included by assuming that all 17 measurements had a concentration of 0.4 μg/m^3^.

#### 3.1.3 West Virginia area specialized silica products study

Richards and Brozell (16) conducted a one-year duration ambient RCS sampling program at the silica production facility in Berkeley Springs, West Virginia. This plant produces a silica product smaller than frac sand. Due to the configuration of the plant located in a valley bounded by steep hills, the downwind sampler had to be located very close to the processing equipment. The mean measured ambient RCS concentration for the downwind site was 1.8 μg/m^3^, and the 95% UCL average was 2.1 μg/m^3^. This plant is not representative of typical frac sand plants, construction sand plants, or aggregate facilities. However, these data, in addition to the Wedron and Maiden Rock data, represent the downwind fenceline concentrations for large mineral industry plants and facilities located in very hilly terrain.

#### 3.1.4 Minnesota area frac sand sampling programs

The Minnesota Pollution Control Agency (11–13) operated RCS sampling programs in the city of Winona, at an agricultural-oriented site in Stanton, and near to mineral industry facilities termed Titan and Shakopee. The data compiled at the Titan (12) and Shakopee (13) frac sand operations used RCS samplers that had LOQs ranging from 1 to 1.3 μg/m^3^, which is more than a factor of 3 above the LOQ for the Upper Midwest and West Virginia data. The Titan and Shakopee data have LOQs exceeding criteria 4 listed previously and have been set aside in determining the typical RCS levels around mineral industry sources.

The MPCA sampling program at Winona (11) was conducted on the roof of a building located in town and close to a frac sand transload operation (23). Jordan Sands in Kasota, Minnesota conducted a three-year sampling program at upwind and downwind sampling locations around the frac sand plant.

All of the RCS data compiled in these MPCA studies were well below the OEHHA REL, and most were close to background RCS concentrations. An ambient RCS sampler operated by MPCA located at an agricultural site in Stanton (12) during the same time period as the sampling program at Winona reported higher RCS concentrations than at the Winona urban site located near a frac sand trans-load facility.

The MPCA data have been converted to a consistent basis with the Upper Midwest studies and a West Virginia study by converting the non-detect values to the LOQ/2. The mean RCS concentrations in the MPCA studies listed in Table 2 ranged from 0.17 to 0.24 μg/m^3^. The 95% upper confidence level of the mean ranged from 0.24 to 0.31 μg/m^3^.

**Table 2:** Comparison of mean and 95% UCL mean downwind concentrations in various RCS sampling programs at typically sized mineral industry facilities.

Facility	Organization and authors	Number of 24-h samples	Mean RCS, μg/m^3^	95% UCL^1^ mean RCS, μg/m^3^	Notes
Typical size mineral industry sources					
Upper Midwest area not including Wedron and Maiden Rock (data from 11 facilities listed in Table 1)	Richards and Brozell	3,134	0.25	0.28	Most studies conducted multi-year
Jordan Sands North	MPCA	44	0.22	0.24	Short duration studies and one multi-year study
Jordan Sands South	MPCA	36	0.24	0.31	
Winona	MPCA	48	0.17	0.18	Near trans load facility
Wisconsin area	University of Iowa (Peters et al.)	17	≤0.40	≤0.40.	Maximum values assigned to all 17 of the 48-h samples
Duarte, California (May-Aug. 2006 and Oct.-Dec, 2006)	SCAQMD	33	0.56	0.65	Numerous nearby mineral industry, community and natural sources
Sample number weighted average			0.25	0.28	
Number of 24-h samples			3,312		

^195% UCLs were calculated for nonparametric distributions.^

#### 3.1.5 California area construction sand studies

The South Coast Air Quality Management District (9, 10) conducted a six month duration study of ambient RCS levels at a school in Durarte, California. This study consisted of a single sampling site. Three large sand and gravel quarries and processing facilities located on three sides of the school were within 4 km of the school. The school was located within 1.2 km of the I-210 and I-605 freeways and adjacent to a dry creek bed. Accordingly, this sampling location had an unusually high concentration of both community and mineral industry sources. SCAQMD sampled ambient air at the school on 33 days over a six month period. The average concentration was 0.58 μg/m^3^ if the non-detect values were calculated as the LOQ/2. This RCS concentrations were well below the OEHHA REL but slightly higher than the data from the Upper Midwest sites. This difference could be due to the concentration of possible sources, the background RCS levels, and the summertime sampling period at Duarte.

Richards et al. (19) conducted a limited ambient RCS sampling program in Vernalis and San Diego, California soon after development of the Appendix L-based sampling procedure described in section 2 of this paper. Only three sets of upwind-downwind data were compiled. In accordance with criteria 5 stated earlier, these data are being set aside in determining typical downwind RCS concentrations.

The California Air Resources Board (20) conducted an ambient RCS sampling program in Lompoc, California. The possible sources of concern included a diatomaceous earth processing plant. However, this work pre-dated the OEHHA REL and was limited to crystalline silica in particulate matter with a 50% cut size at 10 micrometers as sampled using EPA reference methods (PM10) particulate matter. No data from the Lompac study have been included based on criteria 1 stated earlier.

Shiraki and Holmen (21) evaluated ambient respirable silica near a sand and gravel facility in Tracy, California. Quartz was found in PM10 samples. The concentrations of PM2.5 were too low for RCS analyses. Due to the lack of data in the particle size range close to PM4, these data are not representative of the PM4 RCS data in the other papers reviewed.

Table 2 provides a summary of the mean and the 95% UCL mean RCS concentrations in the various sets of published studies meeting the criteria stated in Section 2 of this paper.

The downwind RCS concentrations for the various sampling programs had average values ranging from 0.17 to 0.58 μg/m^3^. The 95% UCL of the means ranged from 0.18 to 0.65 μg/m^3^.

### 3.2 Facility upwind RCS concentrations

Many of the studies providing downwind RCS concentrations also included upwind RCS concentration samplers. Upwind background RCS concentration data are relevant to risk assessment studies because variations in the upwind concentrations have a direct impact on the facility’s downwind RCS concentrations.

The upwind data for the various sets of ambient RCS concentration sampling programs in the Upper Midwest studies are summarized in Table 3 to provide a general indication of background concentrations affecting the available data.

**Table 3:** Comparison of mean and 95% UCL mean downwind concentrations in various RCS sampling programs at large mineral industry facilities and/or facilities located in very hilly terrains.

Facility	Organization and authors	Number of 24-h samples	Mean RCS, μg/m^3^	95% UCL^1^ mean RCS, μg/m^3^	Notes
Berkeley Springs, West Virginia	Richards and Brozell	61	1.8	2.1	One-year, one downwind location
Maiden Rock, WI	Richards and Brozell	219	1.2	1.5	Two years, one downwind location
Wedron, Illinois	Richards and Brozell	139	1.5	1.8	One-year, one downwind location
Wedron, Illinois	ASTDR	127	0.96	1.2	4-months, five downwind locations
Sample number weighted averages			1.3	1.5	
Number of 24-h samples			546		

^195% UCLs were calculated for nonparametric distributions.^

The mean upwind ambient RCS concentrations in the Upper Midwest studies had a narrow range of 0.16–0.25 μg/m^3^. The mean RCS concentration was 0.20 μg/m^3^. The weighted average 95% UCL was 0.22 μg/m^3^. There were 2,194 samples in this data set.

The upwind RCS data in the studies conducted in Minnesota by MPCA, in West Virginia by Richards and Brozell (16) and Pennsylvania Department of Environmental Protection (22) are summarized in Table 4.

**Table 4:** Upper Midwest upwind ambient RCS concentration data.

Facility	Sampling location	Number of 24-h samples	Mean with ND = LOQ/2 μg/m^3^	95% UCL^1^ Mean R CS with ND = LOQ/2 μg/m^3^
Menomonie, WI	Upwind fenceline	61	0.18	0.20
Wedron, IL	Upwind fenceline	128	0.20	0.22
Downing, WI	Upwind fenceline	62	0.24	0.28
Cataract Green, WI	Upwind fenceline	102	0.16	0.18
Cataract Green, WI	Pre-operational	74	0.19	0.22
Maiden Rock, Community WI	Upwind	218	0.22	0.24
Chippewa Falls, WI	Upwind fenceline	386	0.18	0.19
DS Mine, WI	Upwind fenceline	437	0.18	0.19
S&S Mine, WI	Upwind fenceline	372	0.25	0.29
DD Mine, WI	Upwind fenceline	354	0.18	0.19
Sample number weighted average			0.20	0.22
Total number of 24-h samples		2,194		

^195% UCLs were calculated for nonparametric distributions.^

The upwind sampling site at Berkeley Springs was approximately one-half mile from the facility fenceline and in a valley across a ridge near the plant. This sampling location operated for 1 year. The average upwind ambient RCS concentration was 0.30 μg/m^3^, and the 95% UCL was 0.37 μg/m^3^. Some lightly traveled unpaved roads were the only known community sources near the sampling location.

The ambient RCS data measured by MPCA in Stanton, Minnesota (11) had average levels of 0.21 μg/m^3^ if the non-detect data published by MPCA are calculated as the LOQ/2 to be consistent with the Upper Midwest data. Stanton is an agricultural area without nearby mineral industry sources. The RCS concentration data in Station exceeded the levels measured by MPCA in the town of Winona close to a frac sand transload facility.

The Pennsylvania DEP measured ambient RCS concentrations at three sites in the town of Tunkhannock (22). All of these data were compiled in anticipation of a possible frac sand facility in the future. The average ambient RCS levels averaged 0.17 μg/m^3^ if the non-detect data were assigned a value of 0.16 μg/m^3^.

The upwind data summarized in Tables 4, 5 indicate background RCS levels that ranged from mean values of 0.16–0.28 μg/m^3^ and 95th confidence interval values of 0.18–0.37 μg/m^3^. These ranges are similar and slightly lower than the ranges of the downwind data at most of the sites near mineral industry sources. Accordingly, in a risk assessment evaluation of a specific mineral industry source, an accurate measurement of the prevailing background levels of RCS is needed. The background concentrations are related to sources including, but not necessarily limited to (1) unpaved roads, (2) agricultural sources, (3) vegetation burning, (4) construction activity, (5) other nearby industrial sources, and (6) global dust transport.

**Table 5:** Minnesota, West Virginia, and Pennsylvania upwind and background ambient RCS concentration data.

Facility	Sampling location	Number of 24-h samples	Mean with ND = LOQ/2 μg/m^3^	95% UCL^1^ mean RCS with ND = LOQ/2 μg/m^3^
Stanton, Minnesota (MPCA)	Agricultural area	55	0.21	0.24
Berkeley Springs, West Virginia (Richards and Brozell)	Upwind site	61	0.30	0.37
Tunkhannock, Pennsylvania (PA DEP)	Pre-operational sampling sites, 1, 2, and 3	103	0.17	0.19
Sample number weighted average			0.22	0.25
Total number of samples		217		

^195% UCLs were calculated for nonparametric distributions.^

## 4 Conclusion

Relatively recent adaptations of the well-accepted EPA PM2.5 monitoring method specified in 40 CFR Part 50, Appendix L to measure PM4 (respirable) particulate matter have provided an accurate and convenient means to obtain samples for ambient RCS. The ambient RCS data can be compared with the extensive health effects information obtained using occupational exposure data.

The available RCS concentration data provided by a variety of researchers are similar and were obtained at a diverse set of facilities under a wide variety of weather conditions. Sufficient ambient RCS data are available to provide a basis for risk assessment.

If there are community health concerns at the RCS concentrations lower than the 0.31 μg/m^3^ limit of quantitation used many of the studies summarized in Tables 1, 2, new sampling procedures that provide higher 24-h sample volumes and/or more sensitive analytical techniques would be needed to provide accurate community exposure data.

### 4.1 Downwind RCS concentrations

The downwind RCS data summarized in this paper are all in the range of 10% to approximately 50% of the OEHHA REL. The reported RCS concentrations reported by various researchers are similar despite differences in sampling equipment, sampling location characteristics, terrains, and weather conditions.

Based on the 95% UCL mean data summarized in Table 2, the authors recommend a mean downwind ambient RCS concentration of 0.28 μg/m^3^ to represent typical concentrations beyond the fencelines of frac sand, construction sand, and aggregate facilities. Based on the Wedron, Berkeley Springs, and Maiden Rock data sets summarized in Table 3, a 95% UCL mean concentration of 1.5 μg/m^3^ adequately represents the downwind RCS concentrations that exist around very large sources with samplers very close to process areas and/or facilities located in hilly areas with limited emission dispersion.

The RCS concentration data measured in these studies around mineral industry sources are similar to the urban area average concentration of 0.36 μg/m^3^ reported by Davis in his 1984 study or PM2.5 particulate matter sampled using dichotomous samplers.

### 4.2 Upwind RCS concentrations

The upwind data (background) RCS concentrations summarized in Tables 4, 5 ranged from mean values of 0.20–0.28 μg/m^3^ and 95th UCL mean values of 0.22–0.31 μg/m^3^. These ranges are similar and slightly lower than the ranges of the downwind RCS data at most of the sites near mineral industry sources.

Accordingly, in a risk assessment evaluation of a specific mineral industry source, an accurate measurement of the variability of the background levels of RCS is very helpful.

Based on the data summarized in Tables 4, 5, the authors recommend a mean background ambient RCS concentration of 0.22 μg/m^3^ to represent typical concentrations upwind of the fencelines of frac sand, construction sand, and aggregate facilities.

## Footnotes

^1^The Partisol 2000i is now supplied by Thermo Electron.
